# Complete Genome of *Enterobacteriaceae* Bacterium Strain FGI 57, a Strain Associated with Leaf-Cutter Ant Fungus Gardens

**DOI:** 10.1128/genomeA.00238-12

**Published:** 2013-02-21

**Authors:** Frank O. Aylward, Daniel M. Tremmel, David C. Bruce, Patrick Chain, Amy Chen, Karen Walston Davenport, Chris Detter, Cliff S. Han, James Han, Marcel Huntemann, Natalia N. Ivanova, Nikos C. Kyrpides, Victor Markowitz, Kostas Mavrommatis, Matt Nolan, Ioanna Pagani, Amrita Pati, Sam Pitluck, Shweta Deshpande, Lynne Goodwin, Tanja Woyke, Cameron R. Currie

**Affiliations:** a Department of Bacteriology, University of Wisconsin-Madison, Madison, Wisconsin, USA; b Department of Energy, Great Lakes Bioenergy Research Center, University of Wisconsin-Madison, Madison, Wisconsin, USA; c Los Alamos National Laboratory, Biosciences Division Genome Science, Los Alamos, New Mexico, USA; d U.S. Department of Energy (DOE) Joint Genome Institute, Walnut Creek, California, USA

## Abstract

The *Enterobacteriaceae* bacterium strain FGI 57 was isolated from a fungus garden of the leaf-cutter ant *Atta colombica*. Analysis of its single 4.76-Mbp chromosome will shed light on community dynamics and plant biomass degradation in ant fungus gardens.

## GENOME ANNOUNCEMENT

The *Enterobacteriaceae* bacterium strain FGI 57 was isolated in 2009 from a fungus garden of the leaf-cutter ant *Atta colombica* on Pipeline Road in Panama. Leaf-cutter ants are dominant herbivores in the Neotropics that derive nutrition from plant biomass by culturing specialized fungus gardens on foliar material (1). Rather than consuming the plant material directly, they use it as manure to cultivate specialized fungus gardens composed primarily of the obligate fungal symbiont *Leucoagaricus gongylophorus* (2). Recent work has shown that bacteria within the family *Enterobactereaceae* and genus *Pseudomonas* also inhabit fungus gardens (3–5), and metaproteomic investigations of these environments have identified several strain FGI 57 proteins (3), suggesting that this bacterium is an active member of the fungus garden community.

The genome of *Enterobacteriaceae* bacterium strain FGI 57 was generated at the U.S. Department of Energy (DOE) Joint Genome Institute (JGI) using a combination of Illumina (6) and 454 (7) technologies. For this genome we constructed and sequenced an Illumina GAii shotgun library (46,058,996 reads) totaling 4,605.9 Mb together with both a 454 Titanium standard library (110,558 reads) and a paired-end 454 library (269,554 reads, average insert size of 7 kb) totaling 110.8 Mb. Assemblies were generated using a combination of Newbler, version 2.3-PreRelease-6/30/2009; Velvet, version 1.0.13 (8); and parallel Phrap, version SPS-4.24 (High Performance Software, LLC). Consed was used in the finishing process (9–11). Illumina data were used to correct potential base errors and increase consensus quality using the software Polisher developed at JGI (A. Lapidus, unpublished). Possible misassemblies were corrected using gapResolution (Cliff Han, unpublished) or Dupfinisher (12) or by sequencing cloned bridging PCR fragments. Gaps between contigs were closed by editing in Consed, and by PCR and bubble PCR (J.-F. Cheng, unpublished data) primer walks. A total of 38 additional reactions were necessary to close gaps and to raise the quality of the finished sequence. All general aspects of library construction and sequencing performed at the JGI can be found at http://www.jgi.doe.gov/.

The complete genome of the *Enterobacteriaceae* bacterium strain FGI 57 comprises a single circular chromosome of 4.76 Mb with an average of 228.5-fold coverage and a G+C content of 54.0%. Annotation of the finished chromosome was performed using the Integrated Microbial Genomes-Expert Review (IMG-ER) pipeline (13). A total of 9 copies of 16S rRNA, 86 tRNAs, and 4,548 protein-coding genes were identified in this way. This bacterium was originally classified in the genus *Cronobacter*, but Basic Local Alignment Search Tool (BLAST)-based comparison (14) of its 16S rRNA genes with those of other *Enterobacteriaceae* indicate that *Raoultella planticola, Kluyvera georgiana*, and *Citrobacter freundii* all have high nucleic acid identity (all 98%). Phylogenetic analysis of the 16S rRNA genes using FastTree (15) failed to resolve the placement of this bacterium, suggesting that strain FGI 57 may represent a novel group within the *Enterobacteriaceae*. The availability of the complete genome of this bacterium will facilitate future investigations of the symbiotic microbial community residing in leaf-cutter ant fungus gardens.

### Nucleotide sequence accession number.

The complete genome sequence of *Enterobacteriaceae* bacterium strain FGI 57 has been deposited at DDBJ/EMBL/GenBank under the accession number CP003938.
